# A Study of a Parametric Method for the Snow Reflection Coefficient Estimation Using Air-Coupled Ultrasonic Waves

**DOI:** 10.3390/s20154267

**Published:** 2020-07-31

**Authors:** Krzysztof Herman, Tadeusz Gudra, Krzysztof Opieliński, Dariusz Banasiak, Tomasz Budzik, Nathalie Risso

**Affiliations:** 1 Department of Electrical and Electronics Engineering, University of the Bio Bio, Concepción 4081112, Chile; nrisso@ubiobio.cl; 2 Department of Acoustics and Multimedia, Faculty of Electronics, Wroclaw University of Science and Technology, 50-370 Wroclaw, Poland; tadeusz.gudra@pwr.edu.pl (T.G.); krzysztof.opielinski@pwr.edu.pl (K.O.); 3 Department of Computer Engineering, Faculty of Electronics, Wroclaw University of Technology, 50-370 Wroclaw, Poland; dariusz.banasiak@pwr.edu.pl; 4 Department of Earth Sciences, University of Silesia, 41-200 Sosnowiec, Poland; tomasz.budzik@us.edu.pl

**Keywords:** air-coupled ultrasound, snow parameters, digital signal processing

## Abstract

In this paper, a method for estimating snow pressure reflection coefficient based on non-contact ultrasound examination is described. A constant frequency and air-coupled ultrasound pulses were used in this study, which incorporates a parametric method for reflected energy estimation. The experimental part was carried out in situ in the Antarctic, where the snow parameters were measured along with meteorological data. The proposed method represents a promising alternative for estimating the snow-water equivalent, since it uses a parametric approach, which does not require measurements of absolute values for acoustic pressure.

## 1. Background of the Study

Snow deposition or snowpack is one of the most important elements of Earth’s water cycle, widely discussed in [1,2]. The ongoing climate change has worldwide effects; however major perturbations are observed in the Arctic and Antarctic regions, where the majority of the glaciers are located. There is plenty of research related to glaciers and their influence on climate, both in local and global scale. The mainstream message is that the glaciers are retreating and losing their mass; however each year during the winter season the white fields of snow and ice are growing because of the snow accumulation. In fact, what matters, is the glacier mass balance, where the snow deposition and ablation are essential factors.

In general, methods for snow cover evaluation can be classified in terms of scale, as methods applied globally and locally. The first ones use mainly remote sensing based on satellite techniques, where snow-ice cover properties are estimated using electromagnetic waves and electrical characteristics of snow [3,4]. Methods used locally are usually based on manual measurements and observations, which are characterized by their high precision and the variety of snow parameters that can be measured [5]. Since snow is a part of water circulation process, one of its most important parameters is the Snow-Water Equivalent (SWE), which is associated with freshwater capabilities of the deposited snow volume [6]. Currently, besides satellite-based methods, some automatic solutions are being used to evaluate selected parameters of the snow cover. The first method is based on ultrasonic measurements of the snow accumulation, which relies on sensor-snow distance measurements. The second one is photogrammetry [7] combined with image processing techniques [8]. The third one uses GPS-based measurements, where not only altitude-based methods are used, but also the phase difference of the GPS carrier signal is applied [9,10] to estimate GPS position and therefore measure snow accumulation. The Snow-Water Equivalent is derived from the mass conservation principle of a snow sample which transforms into water when melting over a normalized area: $m_{snow}=m_{water}$. Thus, the SWE represents the height of water melted from a snow sample of height HS and density $\rho_{snow}$ over the same area.

From the acoustics point of view, the air–snow border can be considered to be an interface constituted between two different media characterized by their acoustic impedance. Although the gas medium can be considered to be a homogeneous and linear one, snow acoustics characterization implies certain difficulties, related to the snowflakes shape, size, snowpack water content, snow hardness and snow density. Changes in these parameters affect sound energy transmission and reflection. There are various models for acoustic wave propagation and attenuation in snow [11,12,13]; however, the majority of the developed theories were applied in audible frequency range. An interesting study on snow acoustics properties was presented in [14], where the author applied porous media theory to model propagation and attenuation of sound waves in snow. Changes in sound velocity vs. snow density and porosity were modeled as well as sound attenuation, as a function of porosity. Both, snow density and sound velocity changes affect the snow acoustic impedance. In the article [15] some laboratory results of pressure reflection and transmission coefficients measurements were presented. It was shown that there exists a correlation between snow density and pressure reflection coefficient, which can be used to evaluate not only snow depth, but also snow density, using ultrasonic waves, thus improving accuracy of the SWE estimation.

This article is organized as follows: the first section describes different approaches used in these types of studies and methods used in field measurements. The second section contains theoretical background and description of the proposed method. The third section addresses measurement setup, DSP methods applied in the offline analysis, and details of a field experiment performed in Antarctica. The results obtained prove a correlation exists between snow accumulation and acoustics reflection coefficient.

## 2. Theoretical Background of the Proposed Method

**Figure 1:**
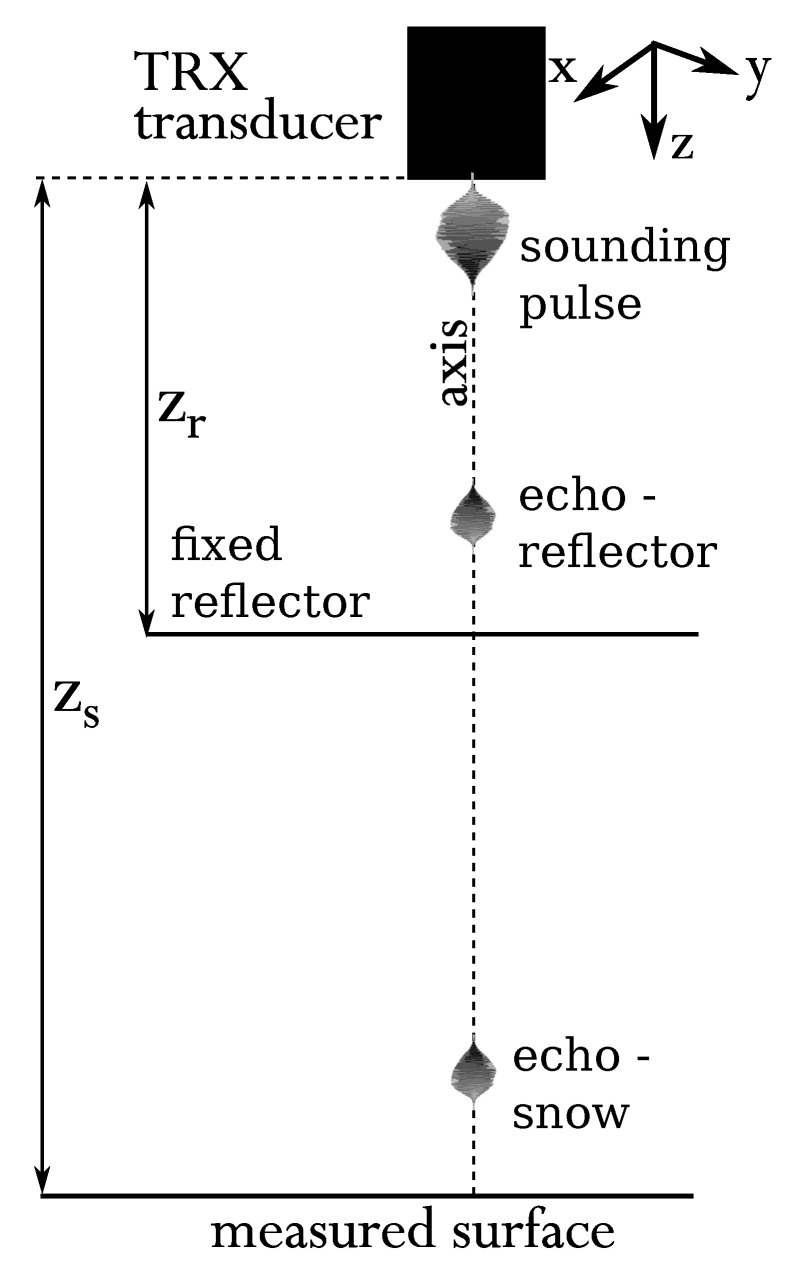
Parametric method for pressure reflection coefficient estimation. The directivity characteristic of the sound transmitter-receiver is assumed to be symmetric with respect to the xy axis.

As it can be found in the articles [16,17,18], the most accurate method for snow depth measurement using ultrasonic waves is the pulse-echo method, combined with parametric compensation of sound velocity changes along the temperature variations. The parametric method for distance estimation, uses a reflector positioned at a fixed distance $z_{r}$ that provides temporal reflector time of flight (TOF) $t_{r}$, which then is used to estimate the unknown target distance $z_{s}$ as shown in Equation (1)

(1) $$z_{s}=\frac{t_{x}}{t_{r}}z_{r},$$

where $z_{s}>z_{r}$ and $t_{x}$ corresponds to the TOF for a wave reflected from the snow surface, see the Figure 1. Assuming there is no temperature gradient between the measurement surface (snow cover) and the sensor head, the measure is sound velocity (air temperature) independent.

The presented method for pressure reflection coefficient estimation shares a similar idea for measurements, where the sound energy reflected from a fixed, known geometric target is used as a reference for estimation of the unknown sound energy reflected from the investigated surface. The pressure reflection coefficient $R_{p}$ is defined as shown in Equation (2) [19]

(2) $$R_{p}=\frac{p_{r}}{p_{i}}\cdot 100\;\left[\%\right]$$

and it can be interpreted as a temporal ratio of the RMS value of the reflected sound pressure wave $p_{r}$ with respect to the RMS value of the incident sound pressure wave $p_{i}$. Due to the methodology applied in this study, the reflection coefficient will be considered.

The proposed method incorporates ultrasonic transducer operating in a pulse-echo mode, where the time of flight (TOF) and energy of received pulses can be estimated using the range equation described in the paper [20] and widely discussed in the article [21]. Assuming a clear, along the axis sound path between transducer and snow surface, the snow echo level $EL_{s}$ is given by Equation (3)

(3) $$EL_{s}=SL-2TL_{s}+20\log_{10}\left(R_{p}\right)\;\left[\mathrm{dB}\right]$$

where SL corresponds to the source level in [dB], $TL_{s}=20\log_{10}\left(z_{s}/d_{0}\right)+2\alpha z_{s}$ denotes total transmission losses for the spherical wave propagation in [dB], α is the coefficient of ultrasound attenuation in the air in [dB/(m·Hz)], and $d_{0}$ is the near field of the ultrasonic transducer. The attenuation in the air α is a function of environmental conditions such as air temperature $T_{c}$, air humidity $R_{h}$, and absolute atmospheric pressure $P_{0}$. Thus $\alpha =f(T_{c},R_{h},P_{0})$.

Considering the sound wave reflection from the reflector positioned at a fixed distance $z_{r}$, the reflector echo level $EL_{r}$ can be described using Equation (4)

(4) $$EL_{r}=SL-2TL_{r}+TS\;\left[\mathrm{dB}\right]$$

where $TL_{r}=20\log_{10}\left(z_{r}/d_{0}\right)+2\alpha z_{r}$ represents transmission loses ($d_{0}$ = 1 m) and $TS=10\log_{10}\left(I_{r}/I_{i}\right)=20\log_{10}\left(p_{r}/p_{i}\right)$ is called target strength [22], which corresponds to the particular object reflectivity. The parameter TS is commonly used in hydrolocation sonar systems and it also has its analog in radio location radar technology, where it is known as a RCST (*Radar Cross Section*) [23]. According to the book [24], the target strength for a cylinder with infinite length $TS_{I}$ and finite length $TS_{F}$ can be calculated using Equation (5)

(5) $$\left.\begin{array}{cccc} TS_{I} & =10\log_{10}\left(\frac{ar}{2r_{1}}\right) & \; & ka>>1,\;r>a,\\ TS_{F} & =10\log_{10}\left(\frac{9\pi^{4}a^{4}r}{r_{1}^{3}\lambda^{2}}\right) & \; & ka<<1,\;r>a, \end{array}\right.$$

where *a* denotes cylinder’s radii, *r* is distance between cylinder and source surface, $r_{1}$ = 1 m, k=2π/λ and λ stands for wavelength. In the previously cited article [22] a comparison of theoretical and measured values for TS is presented for different objects which serves as ground base for this study.

Assuming that object TS is small in comparison to $R_{p}$, Equations (3) and (4) can be combined to extract the pressure reflection coefficient $L_{R_{p}}=20\log_{10}\left(R_{p}\right)$ using Formula (6)

(6) $$L_{R_{p}}=EL_{s}-EL_{r}+2\left(TL_{s}-TL_{r}\right)+TS$$

Using Equation (6), reflection coefficient $R_{p}$ can be evaluated from measurements of echo levels from the snow and from the fixed reflector, respectively. Since, in the snow measurement, distance $z_{s}$ is usually not known, it should be also estimated to evaluate the associated transmission losses $TL_{s}$.

## 3. Measurement Setup and Field Experiment Details

**Figure 2:**
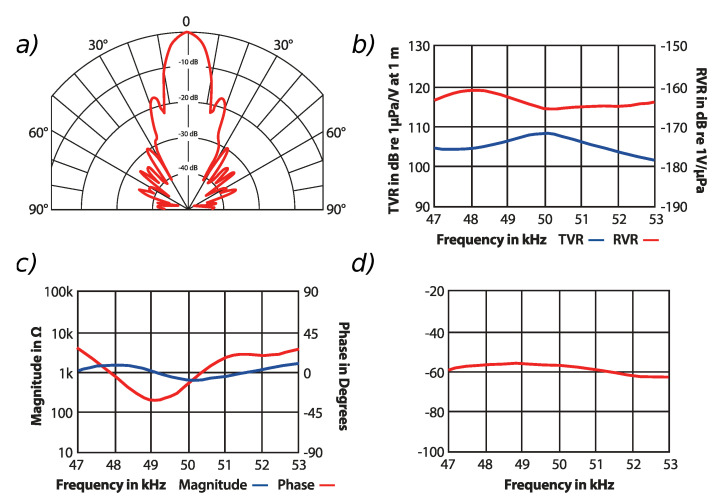
ATK 50 ultrasonic sensor characteristics: (**a**) directivity pattern, (**b**) transmission voltage response TVR and receiving voltage response, (**c**) impedance, (**d**) sum of TVR and RVR. All characteristics based on [25], Copyright Airmar Technology Corporation 2018.

The experiment was carried out using the ultrasonic sensor widely described in [16,17], and modified for performing the full wave signal acquisition. The sensor itself uses the ATK 50 (Airmar) transducer, which is dedicated to operating at 50 kHz ± 4% center frequency in a pulse-echo mode. The sensor characteristics are presented in the Figure 2.

**Figure 3:**
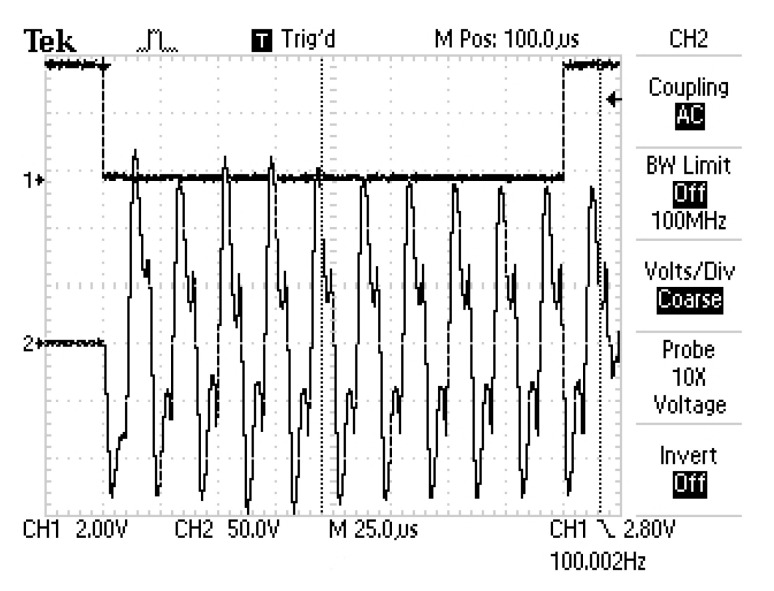
High voltage measured at the transducer input when the pulse is generated.

The transducer radiates the ultrasonic beam using radial vibration of a piezoelectric disc matched to the gaseous medium by a quarter wavelength matching layer. To maximize the source level SL the transducer was excited by a high voltage square wave packet of amplitude at the level of hundreds of volts, which can be observed in Figure 3.

**Figure 4:**
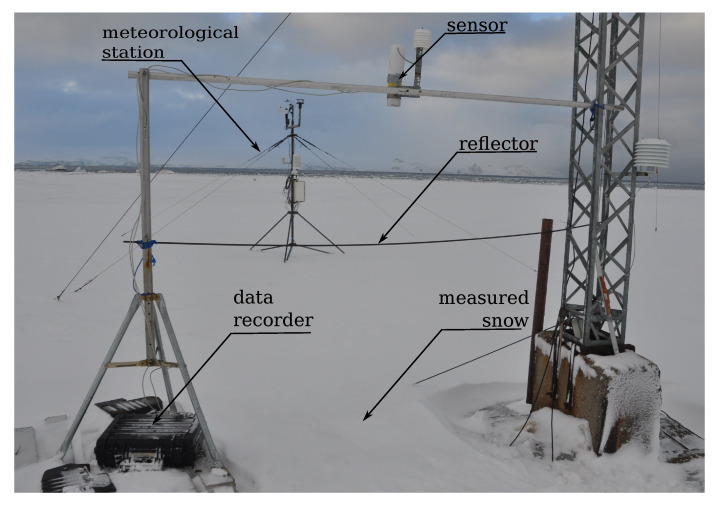
Measurement setup. In first plane: ultrasonic sensor with reflector installed. In second plane: Automatic Weather Station.

A 250 kHz, 16-bit signal acquisition module was added to the sensor to sample the sensor signal and store it on an SD card as “.wav” files for further offline analysis. The sensor was placed at approximately 2 m over the ground, while as a reflector, a metal bar of a radii 4 mm was used. The reflector was situated at a fixed distance $z_{r}$ of 82 cm below the sensor head. The sensor was configured to perform 12 unit measurements separated by a time interval of 30 s. Due to the SD card capacity limitation of 16 GB, only 8 h of a continuous data logging could be performed at once. The measurement setup was installed next to a Campbell Automatic Weather Station, which was logging environmental data such as: air temperature $T_{c}$, relative air humidity $R_{h}$, air pressure $P_{A}$, wind speed $W_{S}$, wind direction $W_{D}$, shortwave radiation $K_{C}$. The field studies were carried out in the vicinity of the Polish Antarctic Station “Arctowski”, in King George Island, Antarctic, during the time period between 2 August 2014–12 August 2014. Table 1 characterizes in detail the environmental measurement setup shown on the Figure 4.

Besides automatic sensor measurements, some manual observations of snow cover were made to supplement the experiment database. Observations were performed according to Classification for Seasonal Snow on the Ground [5] instructions and methodologies. Snow density was captured using a digital balance with resolution of 0.1 °C and a fixed, thin wall tube of volume 201 cm^3^. Additionally, snow grain size density, grain shape, hardness, wetness, cover type and roughness were registered.

## 4. Signal Processing Methods

As mentioned in Section 1 to estimate the reflection coefficient, the ratio $p_{r}/p_{i}$ must be calculated. Here $p_{x}$ corresponds to the root mean square value, which was estimated using a finite number of samples using the expression (7)

(7) $${\hat{p}}_{x\mathrm{RMS}}=\sqrt{\frac{1}{N}\sum_{k=n_{0}}^{n_{0}+N}p^{2}\left(k\right)}$$

where p(k) corresponds to the current sample value within the observation window. The index $n_{0}$ represents the beginning of the signal, and it was evaluated using estimation of the maximum of the autocorrelation function defined as (8). The offline analysis was performed using SciLab computational environment. To capture the entire signal (TX pulse, reflector echo, snow echo), time series were read by sections of 7 k samples. Within the frames a subframe was applied to determine the signal presence and the TX pulse position.

(8) $${\hat{p}}_{c}\left(n\right)=\frac{1}{n_{max}-n_{min}}\sum_{k=n_{min}}^{n_{max}}p\left(n\right)p\left(n+k\right)$$

**Figure 5:**
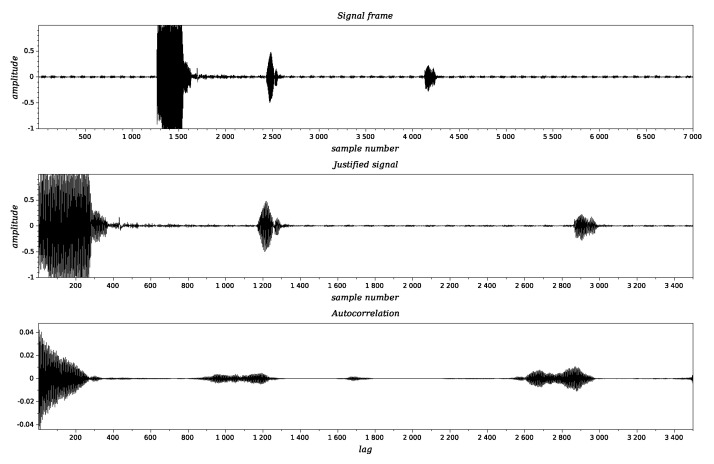
Subsequent signal processing stages: original signal (**top**), the same signal justified to the beginning of the transmitting pulse (**middle**), autocorrelation function of the signal (**bottom**).

The subsequent signal processing stages are shown in Figure 5.

Once detected the signal, autocorrelation was calculated and maximums for the autocorrelation function were evaluated to detect the echo signals. This method allows measurement of the time of flight accurately, thus permitting to calculate transducer-reflector time $t_{r}$, and transducer-snow distance by using $t_{s}$. Once detected, the echo signals were framed, and the RMS value was calculated for each one. Since the measurements were performed taking 12 unit measures of 30 s each, the mean value of each parameter (after rejection of extreme values) was calculated. Moreover, to adjust the time interval of the ultrasonic sensor measurements with the meteorological data (sampled each minute), additional 2 point moving average and downsampling were applied.

## 5. Field Study Results

Because the fact that data sampling period was not continuous, the analysis of the obtained results considers two approaches: generalized results for whole time period and results refereed to a particular period of observation. The complete analysis was performed using R language.

In Table 2 some global descriptive statistics of intermediate parameters estimated according to the methods described in the previous sections are shown.

**Figure 6:**
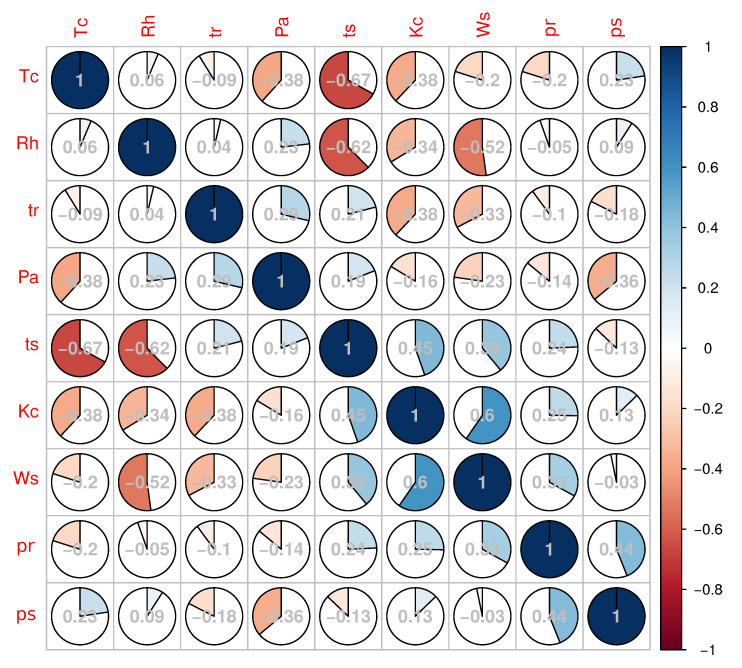
Correlation matrix. The symbols used are interpreted as follows: ambient temperature $T_{c}$, relative air humidity $R_{h}$, time of flight associated with the reflector $t_{r}$, absolute air pressure $P_{A}$, time of flight associated with the snow layer $t_{s}$, shortwave radiation $K_{C}$, wind speed $W_{S}$, sound RMS pressure of the echo associated with the reflector $p_{r}$, sound RMS pressure of the echo associated with the snow layer $p_{s}$.

The overall cross correlation matrix is shown in the Figure 6. It can be observed that there exists a negative correlation between the ambient temperature $T_{c}$ and time $t_{r}$. The Parson’s two-sided correlation test with 95% confidence interval was applied to positively verify this result (t=−45.45, df=3238, *p*-value $<2.21^{-16}$, corr=−0.6241). The same test applied between the ambient temperature and snow layer height, defined as $S_{LH}$ = 2 − 0.8 $t_{s}/t_{r}$ shows very week correlation with a high *p*-value (t=−2.1985, df=3238, *p*-value =0.02799, corr=−0.03860633). Using the same method, an overall correlation between $S_{LH}$ and the reflection coefficient *R* was calculated providing following values t=−35.73, df=3238, *p*-value $<2.21^{-16}$, corr=−0.532.

**Figure 7:**
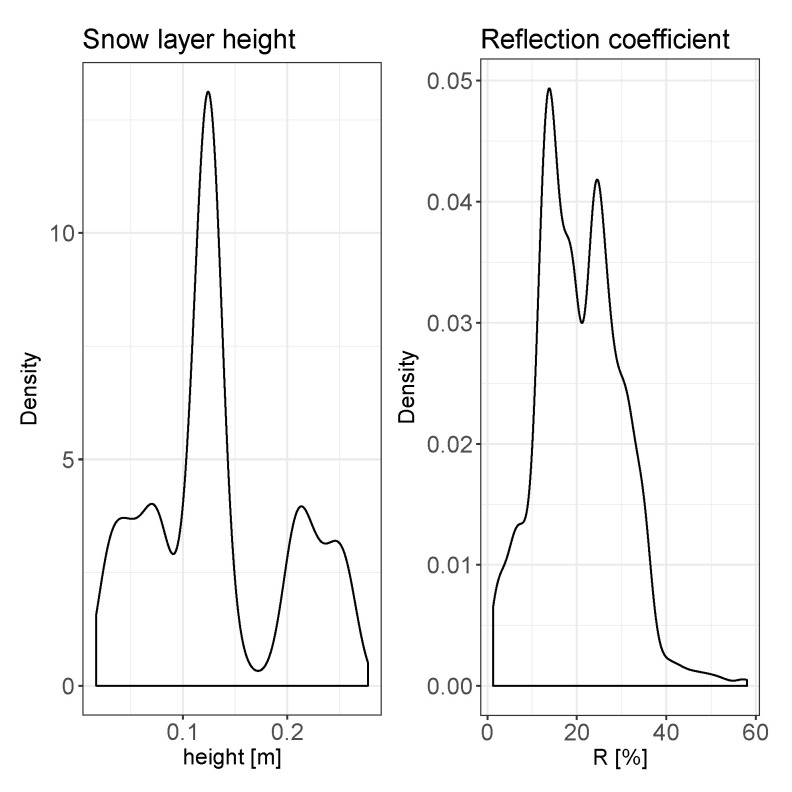
Statistical distribution for two major measurement parameters: snow layer height and reflection coefficient.

**Figure 8:**
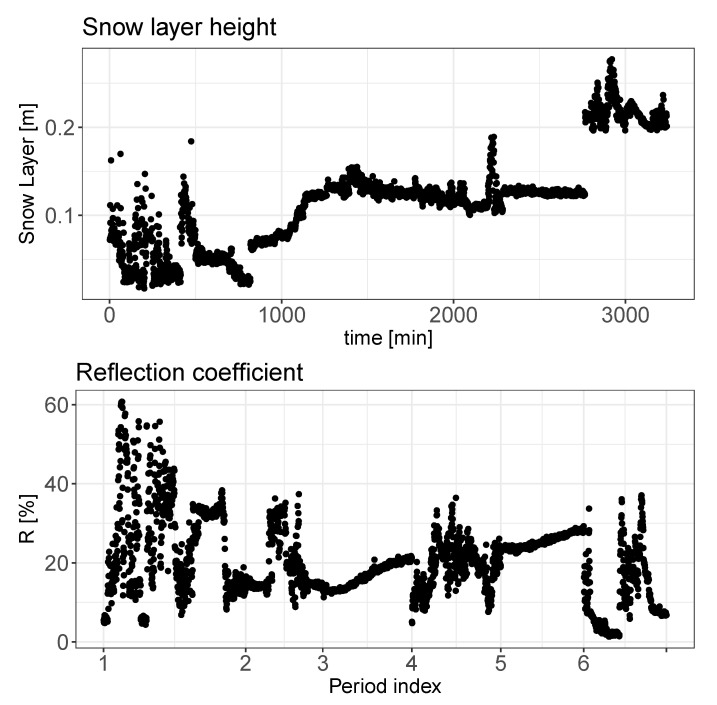
Snow layer height and reflection coefficient as a function of time. The Period index is aligned with the measurement number found in Table 3.

The implementation of the presented method allows estimation of the snow layer height and reflection coefficient. The overall statistical distribution and time dependency of these two parameters are presented in Figure 7 and Figure 8, respectively.

**Figure 9:**
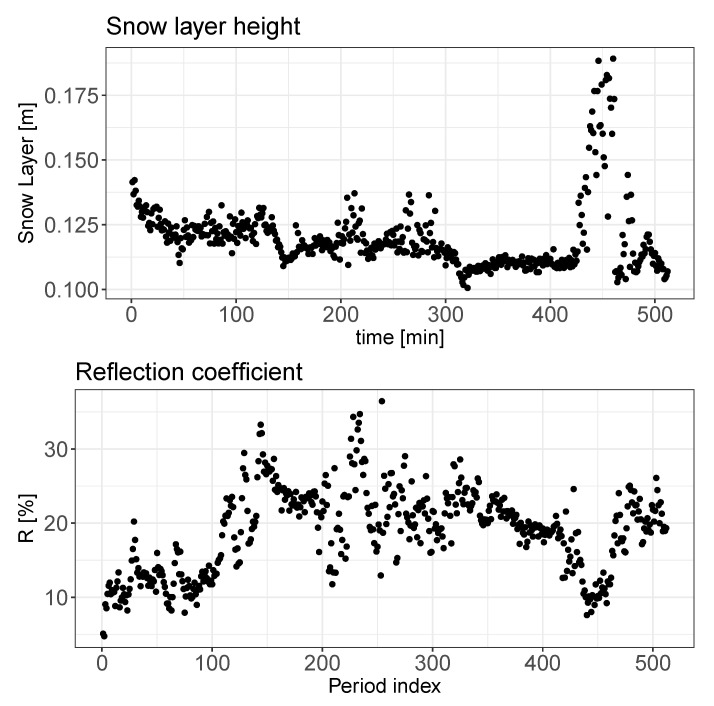
Snow layer height and reflection coefficient as a function of time for a particular, continuous period of 8 h approximately.

In the Figure 9 two time series are presented: the snow layer height and the reflection coefficient estimated using the parametric method proposed.

The manual observations for this period indicate presence of snowfall, where the density of the snow cover was measured manually to be ρ = 86 kg/m^3^, snow deposition of very low hardness, light wavy surface. The snow particles structure characterized as “fine” (0.2–0.5 mm), decomposed and fragmented.

## 6. Discussion

**Figure 10:**
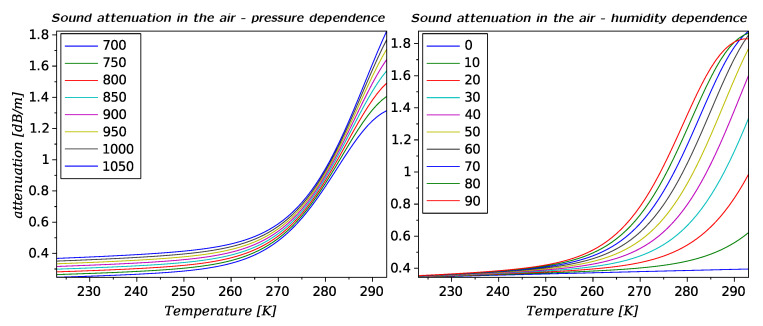
Sound attenuation coefficient for a 50 kHz ultrasonic signal as a function of the absolute temperature for various values of air humidity in % [26].

The results obtained can be divided in to three groups: acoustics data related to the acoustic wave propagation and parameters of the ultrasonic signals, snow physical data where snow density and snow height seem to be the most relevant variables, and the set of meteorological data registered in the vicinity of the experimental setup. The proposed method is based on energy estimation of echo signals registered in situ and processed offline. Comparing the descriptive statistics of the intermediate values presented in the previous section in Table 2, we can observe that: values for times of flight $t_{r}$ and $t_{s}$, which correspond to the echoes reflected from the metal bar and from the snow respectively, are specified by a significant difference between values of variance related to the mean value. Since the parameter $t_{r}$ depends mainly on the temperature variation, changes in temperature should be, and indeed are, reflected in the correlation between value of $t_{r}$ and ambient temperature $T_{c}$. Second time parameter $t_{s}$ varies according to the snow level, which generates a high variance. On contrary, both values for estimated RMS pressure for echoes reflected from the metal bar $p_{r}$, and from the snow $p_{s}$ present a large value of variance with respect to the mean value. This result is surprising since it is assumed that echo level from the fixed bar should have a stable value. Analyzing the correlation matrix, no other significant relation with other variables can be identified so far. In the calculations the fixed value of atmospheric attenuation was assumed to be 0.6 dB/(m MHz), which, analyzing the Figure 10 represents a worst case scenario with respect to a whole range of changes in atmospheric conditions. Analyzing the second pressure parameter, $p_{s}$, and the correlation matrix it can be said that there exists a relation between this parameter and the $t_{s}$ value. The negative correlation comes from the applied model of reflection from the snow presented on Equation (3).

**Figure 11:**
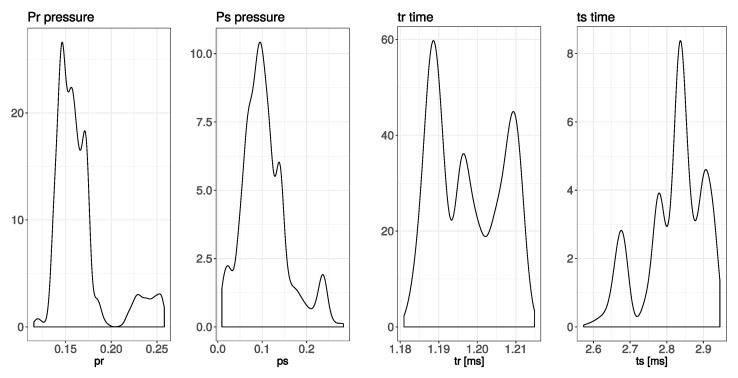
Statistical distribution of the estimated intermediate parameters.

**Figure 12:**
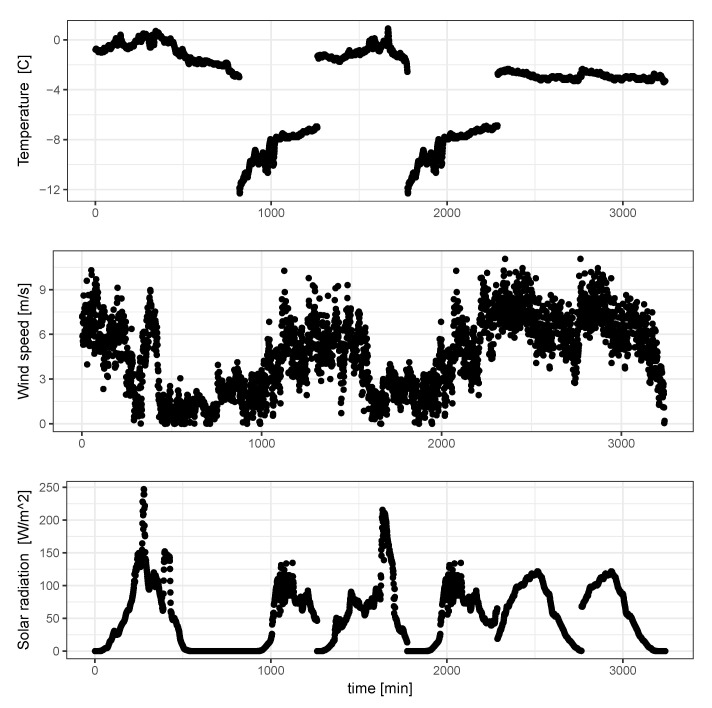
Some meteorological data captured during measurement sessions.

A deeper analysis of the two pressure parameters can be performed taking into account the results of the probability density function presented on the Figure 11, where probability densities of the above discussed parameters are shown. A similarity in the probability distribution for the two pressure parameters can be noticed; however it is supposed that the value of the $p_{r}$ parameter should be almost constant due to the fixed size and position of the metal bar. Analyzing the correlation matrix, it cannot be clearly stated that there exists any dependency of this parameter on the environmental variables presented on the Figure 12. Analyzing Figure 8 and Figure 9along with the correlation value between the snow level height and the reflection coefficient it can be said that snow level height variations affect the value of *R*. Taking into account the laboratory results presented in [17], where a linear dependency between snow density and pressure reflection coefficient was proved, this variation of *R* can be associated with the fresh water properties.

The presented study of the method for the snow reflection coefficient estimation was performed using a single frequency of 50 kHz, due to application of the piezoelectric transducer ATK50, which works in a resonant mode. The sensors based on piezoelectric transducers, take advantage of the resonance, where the signal and the noise are analyzed in a narrow frequency range. Moreover, working at resonance frequency, the transducer reaches its maximum efficiency and sensibility.

An interesting alternative to the resonant, single frequency ultrasonic sensors are devices based on broadband transducers, which can transmit and receive broadband signals. One of the first applications of the air-coupled broadband ultrasonic signals was described in the article [27], where the authors deliver information about time resolution and signal to noise ration improvements due to incorporation of broadband signals in a range of 0.2–1.4 MHz, along with pulse compression technique. Additionally, the applied technique presents some advantages for the estimation of materials properties (thickness, defects) in comparison to the tone-burst method. Going deeper into the details of the above-mentioned method, one can ask: what kind of signals are broadband?. Most broadband waves are either linear frequency sweep of sine waves or short pulses. A comparative study between these signals can be found in [28], where authors compare the quality of results of pulse compression technique for different types of signals: linear sweeps, non-linear sweeps, maximum length sequence pulses (MLS), Golay complementary sequences (GCS). Some of the applications of the broadband signals in examination of materials and biological tissue can be found in [29,30,31]. To close this part of discussion, it is worth to mention that air-coupled broadbands are common in nature [32]. The bats, which are experts in echolocation produce mainly frequency downswept chirps in order to “correlate” at the neuronal level the received echo and to compensate the Doppler effect during flight.

In our article the reflection coefficient was derived for the snow accumulated at the surface, while the snow cover is not isotropic along the vertical direction, which changes the conditions of the acoustic wave propagation. An interesting study was presented in [12,14], where the snow was analyzed as a porous material using the Biots model and micro-imaging in order to determine sound velocity and attenuation in the snow considering it a porous structure.

## 7. Conclusions

In this paper, a method for estimation the snow reflection coefficient in the ultrasonic range was presented. The analysis of real data taken in the Antarctic shows that there exists variation of the reflection coefficient, which depends on the snow cover properties.

Moreover, after the performed study, additional conclusions can be presented:

- Negative correlation was observed and statistically verified between temperature and reflector echo time of flight. There was no correlation between temperature and snow layer height instead, which implies evidence of distance measurement temperature compensation.
- The presented method does not require measurement of the absolute value of the acoustic pressure.
- Because signal processing used was based on framing, the presented method can be easily migrated to a low cost, embedded DSP-based system, to perform real time processing and parameter evaluation.

Although direct relationship between the reflection coefficient and snow density was not analyzed, due to sparse density of the data set, some relationship between snow accumulation and reflection coefficient does exists. The development of a method for snow-water equivalent based on air-coupled ultrasound may be a step towards development of a new sensor which integrates classic ablatometer and snow density sensing.

## Figures and Tables

**Table 1:** Campbell Scientific automatic weather station, situated 2 m above sea level, LAT 62°09′34″ S, LON 58°28′1″ W, characteristics. The acronyms used stand for: $T_{c}$—air temperature, $R_{h}$—air relative humidity, $P_{A}$—air pressure, $K_{C}$—shortwave radiation, $W_{S}$—wind speed, $W_{D}$—wind direction, MI—measurement interval, DL—data logger.

Quantity	Sensor Used	Unit
$T_{c}$	Vaisala HMP155	°C
$R_{h}$	Vaisala HMP155	%
$P_{A}$	Setra 278	Pa
$K_{C}$	Apogee Instr. SP-110	W/m^2^
$W_{S}$	Gill WindSonic	m/s
$W_{D}$	Gill WindSonic	°
MI	-	60 s
DL	Campbell CR 1000	-

**Table 2:** Descriptive statistics of the intermediate parameters.

	$p_{r}$	$p_{s}$	$t_{r}$	$t_{s}$
mean	0.1654	0.1056	1198	2820
min	0.1153	0.00935	1181	2573
max	0.258	0.2833	1215	2945
var	0.000862	0.0026	80.13	6258

**Table 3:** Snow cover manual measurements results. Scales applied correspond to [5] manual. The acronyms stand for: GS-Grain Size, S-Grain Shape, H-Hardness, W-wetness, CT-Cover Type, R-Roughness.

No.	ρ [kg/m^3^]	GS	S	H	W	CT	R
1	104.4	3	2	2	1	1	1
2	108.2	4	1	1	1	4	5
3	123.1	1	1	2	1	1	1
4	85.8	2	2	1	3	11	2
5	175.3	2	2	3	1	4	1
6	102.5	3	3	1	1	1	2

## Abbreviations

The following abbreviations are used in this manuscript:

SWE	Snow-Water Equivalent
TOF	Time of Flight
RCST	Radar Cross Section
SL	Acoustics source level
EL	Echo level
$TL_{s}$	Transmission losses
TS	Target Strength
$T_{c}$	Air temperature
$P_{A}$	Ambient pressure
$R_{h}$	Relative air humidity
$W_{S}$	Wind speed
$W_{D}$	Wind direction
$K_{C}$	Shortwave radiation
